# Navigating the heat: implementation challenges and opportunities for heat alert communication and rural health data infrastructure

**DOI:** 10.3389/fpubh.2026.1801198

**Published:** 2026-04-15

**Authors:** Elizabeth M. Rojo, Hayden B. Bosworth

**Affiliations:** 1 Department of Population Health Sciences, Duke University School of Medicine, Durham, NC, United States; 2 Center of Innovation to Accelerate Discovery and Practice Transformation (ADAPT), Durham Veterans Affairs Medical Center, Durham, NC, United States

**Keywords:** alerts, climate change, heat, implementation science, rural health, surveillance

## Abstract

Extreme heat is the most significant climate threat to public health, disproportionately impacting marginalized groups, including outdoor workers, older adults, and low-income rural populations. While the physiological consequences of heat-related illness—ranging from cardiovascular strain to acute kidney injury—are well-documented, a critical gap remains in the equitable implementation of mitigation strategies. This paper examines North Carolina as a case study due to its proactive leadership in heat-health mitigation, examining the evolution of the state’s Heat Health Alert System and the NC DETECT surveillance platform. North Carolina is well-positioned to pioneer a multi-modal “push” communication strategy, leveraging the ubiquity of smartphone technology and Wireless Emergency Alerts to provide “just-in-time” guidance to high-risk outdoor workers and rural residents. Simultaneously, the state can strengthen its robust surveillance infrastructure by integrating data from non-traditional care sites, such as farmworker clinics, and standardizing occupational data collection. These advancements would transform existing systems into a comprehensive, community-informed model of resilience. By expanding communication modalities and data inclusivity, North Carolina offers a scalable framework for translating meteorological risk into actionable, equitable policy—ensuring that advancements in climate preparedness protect and empower the most vulnerable populations.

## 1 Introduction

Extreme heat is among the most pressing threats to our health and livelihoods of all climate change related disasters. Hence, heat-related illnesses (HRI) are a growing public health concern (1). Across the world, communities most disadvantaged by extreme heat are historically marginalized communities, under resourced and vulnerable populations such as outdoor workers (2), older adults, and low-income rural communities (3). While there is research conducted on the physiological impacts of HRIs, which include the exacerbation of chronic cardiovascular (4) and respiratory conditions (5), adverse reproductive outcomes such as pre-term delivery (6), and the development of acute conditions like acute kidney injury (7), there is a growing need to systematically assess interventions to minimize heat risk. Correspondingly, evidence-based interventions, such as proper hydration, access to cooling spaces, and rest, have been established as effective measures for HRI prevention (8). However, translating this knowledge into effective, population-level mitigation requires examining existing interventions and addressing implementation challenges. We use North Carolina as a case study of a state that has taken proactive steps in heat preparedness and public health response, while contending with escalating climate exposures and structural constraints beyond its control—highlighting both effective practices and opportunities for strengthening equitable implementation and coordination.

North Carolina’s leadership in mitigating climate-related health risks is anchored in two pioneering initiatives: the 2024 statewide launch of the Department of Health and Human Services Heat Health Alert System (HHAS) (9) and the 2004 North Carolina Disease Event Tracking and Epidemiologic Collection Tool (NC DETECT) (10). Together, these systems of climate adaptation, position North Carolina as a model for translating meteorological hazards into actionable public health intelligence. By integrating environmental data with real-time health surveillance, the state has established a foundation for anticipatory response and data-driven decision making.

As with any mature public health innovation, the next phase may want to focus on optimizing reach, equity, and completeness. Specifically, North Carolina’s heat mitigation infrastructure is well positioned to: (1) expand and diversify alert modalities to ensure timely communication and reach of high-risk outdoor workers and rural residents regardless of digital access, and (2) broadening surveillance to include non-traditional care and community settings so that health data more fully capture rural and under-resourced populations. These opportunities for enhancement reflect the natural evolution of effective public health systems and underscore North Carolina’s capacity for continuous improvement. As the state advances from early implementation to refinement and scale, these efforts illustrate how proactive leadership can adapt to emerging climate challenges while maintaining a strong commitment to equity and resilience.

A key implementation opportunity lies in ensuring the equitable and timely delivery of actionable heat warnings across all communities. Nationally, many alert systems rely on communication channels such as email or broadcast media that do not consistently reach high-risk populations, particularly rural residents, outdoor workers, and individuals with limited digital connectivity. This can create a delay between the identification of meteorological risk and the adoption of protective behaviors at the community level. In North Carolina, the Heat Health Alert System currently relies on email-based dissemination. Broadening alert modalities, such as short message service (SMS), community intermediaries, employer-based notifications, and local health or emergency management channels, represents a next phase to extend reach across diverse technological and socioeconomic contexts.

A second challenge—and opportunity for system strengthening—relates to incomplete capture of HRI data, particularly in rural and highly vulnerable communities. While North Carolina has made substantial progress through NC DETECT by integrating emergency department, poison control, and emergency medical services data, gaps remain in settings where care-seeking occurs outside traditional emergency pathways. These gaps may include underrepresentation of cases managed in urgent care, primary care, community health centers, farmworker clinics, correctional facilities, or those self-managed without formal healthcare contact. As a result, surveillance data may underestimate the true burden, timing, and geographic distribution of HRIs, limiting precision in resource allocation and intervention targeting (11). Addressing these gaps—by expanding data sources, enhancing reporting workflows, and incorporating community-based indicators—offers a clear opportunity to further strengthen North Carolina’s capacity for data-informed, equitable heat-risk mitigation.

North Carolina offers a compelling case study given its proactive attention to extreme heat and the opportunity to build on a growing foundation of data and policy innovation. NC is home to the nation’s second-largest rural population (12) and one of the largest farmworker populations, ranking sixth nationally (13), positioning the state as a critical setting for advancing heat-related protections. Recent efforts, include the establishment of a heat stress advisory council by the North Carolina Department of Labor (14). Expanding this work to more fully integrate expertise from the health sector and farmworker-serving organizations would further strengthen the council’s capacity to design community-informed, evidence-based protections. Co-developing risk mitigation strategies with communities most affected by extreme heat represents a high-impact opportunity to enhance relevance, trust, and effectiveness. Addressing current data limitations in rural areas and improving communication strategies beyond email-based approaches will be essential to deepening community engagement, informing policy development, and safeguarding vulnerable populations as extreme heat risks continue to rise.

We examine the NC DETECT and the HHAS as case studies to address implementation challenges and opportunities for heat alert communication and rural health data infrastructure. By drawing from NC’s effort in mitigating and investigating heat-health risk, this perspective seeks to contribute to the broader discussion of effective interventions and heat disease surveillance.

## 2 Implementation opportunity: expanding and strengthening heat alert communication

The North Carolina Heat Health Alert System, developed by the North Carolina Department of Health and Human Services and launched in 2024, represents an important statewide investment in protecting communities from extreme heat. The system enrolled 579 subscribers in its first year, and introduced updated, season-specific Heat Index thresholds in 2025 to better calibrate risk for changing climatic conditions (15).

Building on this foundation, HHAS presents a timely opportunity to enhance reach and equity through expanded communication modalities. Currently, HHAS delivers alerts via email notifications when forecasted temperatures are expected to reach unhealthy levels within selected counties. While effective for many subscribers, expanding beyond a single communication channel could further strengthen the system’s ability to reach populations at highest risk for heat exposure, including older adults, rural residents, and outdoor workers. Of the reported subscribers in 2024, only 7.9% were older adults (65+), 6.6% worked outside, and 2.4% worked in agriculture (9).

North Carolina’s varied digital landscape further underscores the opportunity for innovation. Although broadband access exceeds 98% statewide, rural and low-resource areas—particularly those with large farmworker populations—experience more variable internet access and lower digital literacy (16). Importantly, smartphone ownership remains high across these communities, including among migrant and agricultural workers, even when internet access is inconsistent (17). This pattern creates a promising pathway for complementary mobile health strategies, such as SMS–based alerts, which can deliver timely, passive, and actionable information directly to users. Such approaches are well aligned with “just-in-time” communication principles that support rapid behavioral response during acute heat events.

The current opt-in, county-specific enrollment structure also offers an opportunity to better support mobile and migratory populations. Farmworkers frequently move across counties during the heat season and enabling statewide enrollment options or automated geographic updating—could substantially enhance continuity and reduce user burden. While HHAS already offers alerts in both English and Spanish, simplifying enrollment and delivery mechanisms would further strengthen the system’s accessibility and usability for individuals with dynamic work and living arrangements.

Other state and regional initiatives have successfully implemented multi-modal communication strategies to ensure redundancy and accessibility. For example, the State of New York and the city of Boston, have developed systems that deliver heat alerts via email or phone call with widely accessible SMS text messaging (18, 19). These multi-channel approaches overcome barriers associated with digital literacy and connectivity, ensuring warnings are delivered directly to the individual’s handheld device without the complexity of the signup process or the consistency of a data connection.

A significant opportunity to improve the reach and immediacy of heat alerts lies in leveraging existing national public safety infrastructure. The Wireless Emergency Alerts (WEA) system, launched in 2012, is a public safety tool capable of sending geographically targeted, text-like messages to all devices on participating wireless carriers within an area of imminent threat (20, 21). Critically, WEA alerts are free and require no opt-in from the user, making them ideal for high-risk, time-sensitive events. North Carolina currently utilizes WEA for threats such as flash floods and severe weather, but not for extreme heat advisories. Recognizing extreme heat as an imminent public safety threat, akin to flooding, would allow NC to utilize this ubiquitous, passively received platform to achieve near-universal reach in target areas.

A recent heat event illustrates a valuable opportunity to further integrate public health messaging into existing, high-reach alert infrastructures. In June 2025, during a major heat dome affecting nearly half of the United States, North Carolina’s largest energy provider successfully deployed a text notification to customers requesting reduced energy consumption to support grid stability (22). This timely, large-scale communication demonstrates the feasibility and reach of passive, population-level alert systems during extreme heat events. With modest coordination, such operational alerts could be leveraged to deliver complementary statewide WEA that combine energy conservation messaging—an approach already adopted in states such as California (23)—with clear, actionable public health guidance, including hydration, shade-seeking, and rest. A study looking at the association between heat warnings, mortality and hospital admission in the US found that while there was no evidence that heat alerts lowered the risk of mortality, it did find an elevated risk of HRI-related hospital admission on heat alert days (24), signaling that awareness could lead to care seeking behaviors. During the NC heat dome event of June 2025, NC DETECT reported 1,095 ED visits, approximately 19% of the total 5,748 ED visits reported in summer 2025 (25). Integrating these messages would be particularly impactful for outdoor and other heat-exposed workers who may not be connected to opt-in channels. Expanding toward a multi-modal alert strategy—anchored in universal, passive mechanisms like WEA and reinforced by opt-in SMS and email—offers a practical pathway to operationalize “just-in-time” communication and advance equitable access to life-saving information. Aligning heat-related public health alerts with the same trusted, ubiquitous infrastructure used for other acute threats represents a high-impact opportunity to strengthen population-level heat resilience across North Carolina.

## 3 Implementation opportunity: rural health data infrastructure strengthening

Adequate public health surveillance is the foundation for understanding, quantifying, and mitigating the health impact of extreme heat. While completely attributing extreme heat as the sole cause of an emergency department (ED) visit has its own challenges, including correctly identifying all HRI patients and documenting heat as a cause of death in extreme cases, improving heat surveillance can help better quantify the burden of extreme heat (26, 27).

In North Carolina, NC DETECT serves as a robust, statewide syndromic surveillance platform that plays a central role in monitoring population-level health trends related to extreme heat (28). Drawing primarily from ED data from larger hospital systems and leveraging ICD-10 heat-related diagnoses, NC DETECT provides a critical backbone for real-time situational awareness and public health response. This infrastructure offers a strong foundation upon which more comprehensive HRI surveillance can be built—particularly as climate-related exposures intensify and diversify across communities.

As healthcare delivery patterns evolve, NC DETECT presents an important opportunity to expand its reach across the full rural healthcare ecosystem. Ongoing rural hospital closures and financial pressures on small facilities underscore the growing importance of incorporating data from decentralized care settings that increasingly serve as the first point of contact for rural and vulnerable populations. Community health clinics, migrant and farmworker health centers, and mobile clinics are frequently utilized for clinically meaningful but non-emergent HRI presentations, such as heat cramps, heat edema, dehydration, and early-stage heat exhaustion. In fact, a study of the NC farmworker population in 2013, reported HRI symptoms in 72% of its participants (29) yet it is unclear if any of them sought emergency department care. Integrating data from these settings would enhance NC DETECT’s ability to capture the full spectrum of heat-related morbidity—Expanding surveillance to include these care sites would improve population estimates, strengthen situational awareness, and better inform the allocation of preventive and outreach resources.

NC DETECT also offers a promising platform for advancing more granular analyses of climate-related health inequities. At present, occupational information—an essential determinant of heat exposure risk—is not systematically captured within the surveillance system. This creates a valuable opportunity for modernization. Introducing a standardized, structured occupational field aligned with the Standard Occupational Classification (SOC) (30) system would allow researchers and public health officials to directly link HRI incidence with occupation, geography, and environmental conditions. Such enhancements would enable more precise identification of high-risk workplaces, support geographically targeted interventions, and generate actionable evidence to inform workplace guidance, employer engagement, and regulatory decision-making.

**Figure 1:**
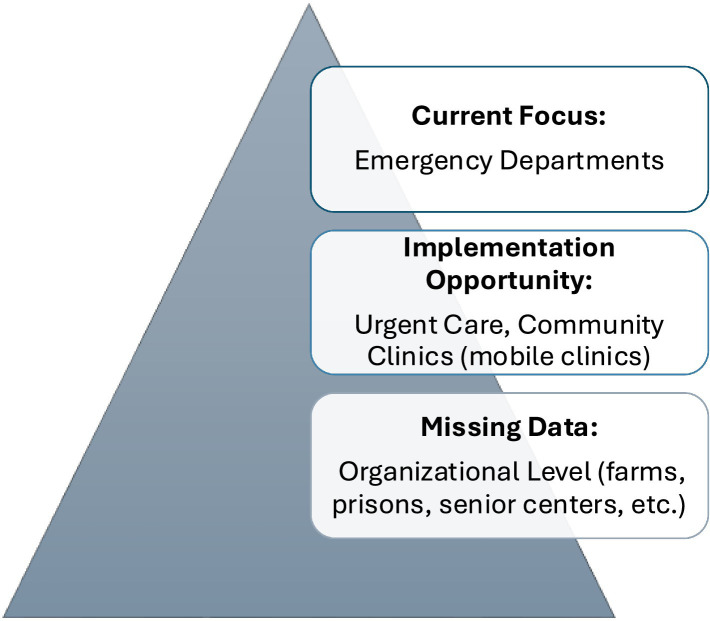
Heat related illness surveillance pyramid.

Beyond technical enhancements, NC DETECT is well positioned to evolve alongside efforts to improve trust and engagement in vulnerable communities. In agricultural and other outdoor occupational settings, structural and policy contexts can influence patterns of healthcare utilization. Strengthening surveillance through complementary, community-informed data collection strategies (i.e., partnerships with trusted clinics, anonymized reporting mechanisms, or aggregate syndromic signals) represents an opportunity to better reflect lived experiences while respecting concerns about safety and confidentiality. Incorporating these perspectives would enhance data completeness, reduce under-ascertainment, and ensure surveillance systems more accurately reflect the true burden of heat exposure across North Carolina. Figure 1 is a conceptual illustration of the gap between HRI cases captured through NC DETECT emergency department surveillance, the additional burden of HRIs treated in non-emergent clinical settings, and the larger community burden of self-managed or organizationally managed cases (i.e., agricultural worksites or correctional facilities) that are not routinely reported because no mandatory reporting policy exists.

Collectively, NC DETECT represents a strong, scalable surveillance infrastructure with opportunities for expansion and refinement. By broadening data sources, enhancing occupational granularity, and further aligning surveillance approaches with rural and high-risk communities, North Carolina can strengthen its capacity to monitor, prevent, and respond to heat-related illness in an increasingly warming climate.

## 4 Discussion: strategic opportunities for implementation excellence

### 4.1 Advancing resilience through actionable policy

Policy interventions are critical to addressing better infrastructure for prevention and surveillance during heat disasters. The momentum for heat-resilient infrastructure is growing, with North Carolina’s early adoption of heat-health surveillance serving as a model for regional coordination. By building upon this foundation, local governments can further refine Heat Action Plans to serve as dynamic roadmaps for community protection, ensuring equitable resource allocation and funding for rural communities (31). While seven states have already pioneered worker protection policies (23), there is an opportunity for North Carolina in creating further inclusive standards. Nevertheless, to safeguard that proper policies and heat action plans are developed while keeping the most vulnerable in mind, we recommend states and local governments to include health professionals from rural and urban settings, as well as outdoor labor representatives—in particular farmworkers—into advisory roles to ensure efforts are inclusive, scientific, expert-based and locally informed.

### 4.2 Optimizing reach through multi-modal communication

North Carolina’s existing communication infrastructure offers an opportunity for rapid, high-impact optimization. By leveraging the WEA system, NC can transition from opt-in models to a universal “push” strategy, delivering life-saving information with the same immediacy as severe weather warnings. This evolution acknowledges the high smartphone penetration among rural and migratory populations. Transitioning to SMS-based delivery reduces “implementation friction” and ensures that linguistic accessibility and “just-in-time” messaging reach outdoor workers and seniors precisely when they are most needed.

### 4.3 Expanding to non-traditional data sources

Improving HRI surveillance requires a multi-pronged strategy focused on both data integration and standardization, particularly in rural and occupational settings where the true burden of illness is currently masked. While data collection in urban settings still requires EDs to modify some of their existing infrastructure or training, documenting for heat-related illnesses will become crucial as scientists continue to explore the true impact of extreme heat across different diseases, populations and geographies. Simultaneously, creating and improving existing digital infrastructure in rural and remote settings to implement and expand on existing larger health systems that feed into surveillance systems such as NC DETECT is paramount.

Equally important is standardizing how to capture work-related ED visits to inform adequate worker protections (32). This standardization in occupational reporting must also account for social and structural barriers to care. Farmworkers, for instance, are less likely to go the ED for heat-related illnesses unless the condition is life-threatening (33). This reluctance is often due to several intertwined factors, including being uninsured or underinsured, lack of transportation or time off to seek medical attention, or a pervasive fear of being racially profiled or targeted by immigration officials. By improving data capture and integrating data from nontraditional data sources such as federally qualified health centers and smaller community clinics, NC DETECT could capture a more comprehensive picture of heat-health trends.

## Data Availability

The original contributions presented in the study are included in the article/supplementary material, further inquiries can be directed to the corresponding author.
